# Future prediction of biogas potential and CH_4_ emission with boosting algorithms: the case of cattle, small ruminant, and poultry manure from Turkey

**DOI:** 10.1007/s11356-024-32666-7

**Published:** 2024-03-05

**Authors:** Ihsan Pence, Kazım Kumaş, Melike Siseci Cesmeli, Ali Akyüz

**Affiliations:** 1 Department of Software Engineering, Bucak Technology Faculty, Burdur Mehmet Akif Ersoy University, Burdur, 15300 Turkey; 2 Bucak Emin Gulmez Vocational School of Technical Sciences, Burdur Mehmet Akif Ersoy University, Burdur, 15300 Turkey

**Keywords:** Animal waste, Environmental effect, Machine learning, Methane, Regression, XGBR algorithm

## Abstract

Animal waste can be converted into a renewable energy source using biogas technology. This process has an impact on greenhouse gas emissions and is a sustainable source of energy for countries. It can reduce the effects of climate change and protect the planet for future generations. Tier1 and tier2 approaches are commonly used in the literature to calculate emissions factors. With boosting algorithms, this study estimated each animal category’s biogas potential and CH_4_ emissions (tier1 and tier2 approach) for 2004–2021 in all of Turkey’s provinces. Two different scenarios were created in the study. For scenario-1, the years 2020–2021 were predicted using data from 2004 to 2019, while for scenario-2, the years 2022–2024 were predicted using data from 2004 to 2021. According to the scenario-1 analysis, the eXtreme Gradient Boosting Regressor (XGBR) algorithm was the most successful algorithm with an *R*^2^ of 0.9883 for animal-based biogas prediction and 0.9835 and 0.9773 for animal-based CH_4_ emission predictions (tier1 and tier2 approaches) for the years 2020–2021. When the mean absolute percentage error was evaluated, it was found to be relatively low at 0.46%, 1.07%, and 2.78%, respectively. According to the scenario-2 analysis, the XGBR algorithm predicted the log10 values of the animal-based biogas potential of five major cities in Turkey for the year 2024, with 11.279 for Istanbul, 12.055 for Ankara, 12.309 for Izmir, 11.869 for Bursa, and 12.866 for Antalya. In the estimation of log10 values of CH_4_ emission, the tier1 approach yielded estimates of 3.080, 3.652, 3.929, 3.411, and 3.321, respectively, while the tier2 approach yielded estimates of 1.810, 2.806, 2.757, 2.552 and 2.122, respectively.

**Graphical Abstract:**

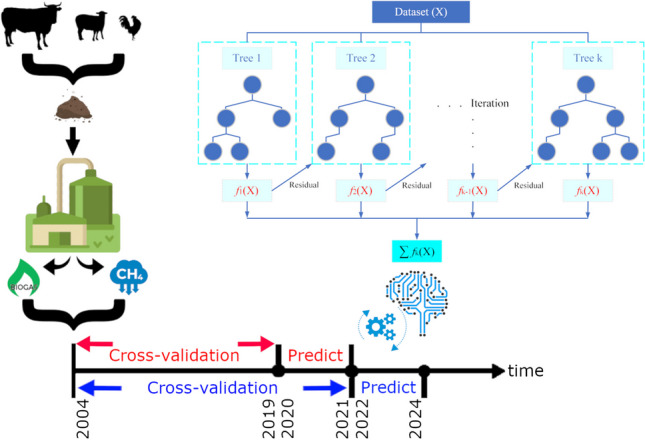

## Introduction

The need for energy worldwide is increasing in line with the population, and this energy demand continues to increase with living conditions and technological developments. Energy demand has increased by 150% in the last 40 years. Energy consumption is thought to have caused an increase of approximately 60% in air pollution. Using clean energy sources is very important to prevent such adverse effects. Greenhouse gases formed during energy production are known as the gases responsible for climate change worldwide. Since these gases’ physical and chemical properties are different, their effects are also different. Due to this global situation, the Kyoto Protocol and the Climate Change Convention were signed in the United Nations Framework Convention on Climate Change (Can 2020; OECD 2015; Senocak and Guner Goren 2022).

The increase in industrial-scale livestock farming worldwide and the resulting animal waste have become significant sources of environmental pollution. Turkey is an important agricultural and livestock country. Livestock farming contributes significantly to agricultural production, rural development, and the economy. Due to Turkey’s growing population and economic development, there is an increasing demand for meat and milk. However, livestock production needs to be controlled. A significant amount of manure is produced in the livestock sector every day. Ensuring hygiene in the livestock sector and proper disposal of the obtained manure are crucial. The irregular disposal of livestock manure and its direct use as fertilizer in agricultural lands are two significant issues in animal manure management in Turkey (Can 2020; Erdogdu et al. 2019; Melikoglu and Menekse 2020; Şenol et al. 2021). A new regulation has been introduced to control animal manure management. This new regulation, effective in the second half of 2021, restricts the direct application of livestock manure to the soil. In addition, livestock farms are held responsible for properly storing animal manure and developing manure management plans (Chandra Manna et al. 2018; Erdogdu et al. 2019; Zaidi et al. 2018). This regulation also encourages the use of animal manure for biogas production as a management strategy. Due to certain restrictions, animal manure is not directly disposed of and is mainly stored in fertilization or storage facilities. Uncontrolled animal manure storage can lead to greenhouse gas emissions and environmental issues such as odor and hygiene problems. Animal manure that does not have proper storage facilities can indirectly contaminate surface water or groundwater. Animal manure contains various microorganisms that can risk animals and humans, causing food contamination and disease outbreaks (Font-Palma 2019; Maroušek et al. 2020; Sun et al. 2021). Therefore, implementing sustainable manure management systems that reduce the environmental risk and allow for the storage, transportation, and use of manure on farms is crucial in many aspects. The livestock and agriculture sectors contribute significantly to Turkey’s greenhouse gas emissions. Therefore, it is essential to target the reduction of greenhouse gas emissions resulting from manure management and find innovative solutions to tackle this significant issue (Erdogdu et al. 2019; Şenol et al. 2021; Sun et al. 2021).

There is a growing trend toward using renewable energy sources to meet the world’s energy needs while reducing environmental damage. There is a growing effort among governments, scientists, and companies to develop sustainable methods of obtaining energy and to enact laws governing the use of such methods. Several sustainable energy sources have been promoted in energy production, resulting in a gradual decrease in the use of fossil fuel-based energy. The shift in electricity generation in European Union member countries is achieved through the promotion of sustainable energy sources and the reduction of reliance on fossil fuels (Gündoğan and Koçar 2022; Karaaslan and Gezen 2022; Ocak and Acar 2021).

This year, for the first time in European Union member countries, 40% of electricity generation came from renewable energy sources, while 34% was derived from fossil fuels. The increasing utilization of renewable energy sources contributes to the transformation aimed at meeting the world’s energy demand while minimizing the impact on the ecosystem. Governments, scientists, and companies are working toward legal regulations to promote energy production and improvements through sustainable methods (Cheng et al. 2021; Erdin and Ozkaya 2019; Pence et al. 2022; Yurtkuran 2021).

This situation demonstrates the effectiveness of policies that reduce dependence on fossil fuels, transition to methods that cause less harm to the environment in energy production, and promote sustainable energy sources. Increasing renewable energy sources reduces greenhouse gas emissions, decreases environmental pollution, and combats climate change (Jones and Moore 2023; Pence et al. 2022; Senocak and Guner Goren 2022).

The anaerobic decomposition of organic materials such as manure, agricultural waste, sewage sludge, and food waste produces biogas. It primarily consists of methane (CH_4_) and carbon dioxide (CO_2_), which can be converted into heat and electricity. Biogas is known as a renewable energy source. It is widely used in Europe due to its advanced technology.

The Intergovernmental Panel on Climate Change (IPCC) Guidelines recommend two general methods for estimating CH_4_ emissions factors: tier1 and tier2. The tier1 method uses default emissions factors to calculate emission factors, while the tier2 method uses country-specific data. Compared to tier1, tier2 is more accurate because it incorporates country-specific information (Dong et al. 2006).

As a clean, renewable energy and fuel with low environmental impact, biogas is used for various purposes such as cooking, lighting, and electricity generation (Khoshgoftar Manesh et al. 2020). It is stated that biogas utilization can reduce greenhouse gas emissions and can be one of the most important energy sources in meeting countries’ energy demands (Usack et al. 2019). Biogas, mainly consisting of 50–70% CH_4_, can be converted into heat and electricity. Biogas is considered a renewable energy source. Renewable energy and related conversion technologies provide an alternative to fossil fuel-derived energy, which is associated with various environmental issues (Heydari et al. 2021; Wang et al. 2021). Recently, it has been stated that photovoltaic/biomass systems are more cost-effective than renewable hybrid systems (Heydari et al. 2023).

In recent years, machine learning (ML)-based models have emerged as promising tools for predicting AD processes (Andrade Cruz et al. 2022). It is possible to estimate and determine biogas production using ML models without understanding the process mechanisms (Tufaner and Demirci 2020). Several studies have utilized ML algorithms such as artificial neural networks (ANN) and random forests (RF), or combinations thereof, applied to biogas processes (Chiu et al. 2022; Gonçalves Neto et al. 2021).

Najafi and Faizollahzadeh Ardabili (2018) studied small-scale biogas production using mushroom compost. This study employed ANFIS (adaptive neuro-fuzzy inference system) and ANN models to predict biogas production based on independent variables. The independent variables considered in the study were the carbon-to-nitrogen (C/N) ratio, reactor temperature (T), and retention time (RT) (Najafi and Faizollahzadeh Ardabili 2018).

De Clercq et al. (2019) developed an ML model to predict biogas output based on waste input. They aimed to improve biogas production in industrial facilities by designing a graphical user interface. The ML model used in the study consisted of logistic regression, support vector machine, random forest, extreme gradient boosting, and *k*-nearest neighbor regression. According to their findings, the *k*-nearest neighbor (KNN) regression model was the most suitable method for the biogas plant, achieving an accuracy of 87% on the test set (De Clercq et al. 2019).

Stolarski et al. (2020) conducted a study on developing bioenergy technologies in Denmark, Germany, Estonia, Finland, Latvia, Lithuania, Poland, Sweden, and Norway. The research focused on assessing the potential of agricultural biomass, manure, and slurry in these countries. The study revealed that Germany and Poland have the highest potential for utilizing agricultural biomass, manure, and slurry for bioenergy production. Furthermore, it was highlighted that Germany, the leading biogas producer, accounted for 92% of all biogas plants in the studied countries (Stolarski et al. 2020).

Elmaz et al. (2020) employed ML methods to predict the outcomes of biomass gasification. Their study utilized four regression techniques: polynomial regression, support vector regression, decision tree regression, and multilayer perceptron. The results showed that the multilayer perceptron and decision tree regression outperformed the other methods regarding prediction accuracy (Elmaz et al. 2020).

Das et al. (2020) utilized farm animal population data from Bangladesh between 2005 and 2018 to estimate greenhouse gas emissions using the 2006 IPCC tier1 approach. They determined that the greenhouse gas emissions from livestock in 2018 amounted to 66.59 Gg/year CO_2_ equivalent. The study further projected that by 2020, the emissions could reach 69.87 Gg; by 2030, 80.62 Gg; by 2040, 94.64 Gg; and by 2050, 113.10 Gg/year CO_2_ equivalent. In 2018, the total greenhouse gas emissions were composed of enteric CH_4_ (44%), manure CH_4_ (3.6%), direct N_2_O (51.5%), and indirect N_2_O emissions (Das et al. 2020).

Almomani (2020) developed an ANN algorithm to model and optimize cumulative CH_4_ production from agricultural solid waste and cow manure (Almomani 2020).

Kim et al. (2020) calculated the impact of digested biogas from organic waste on natural gas and its ability to reduce CO_2_ emissions for two Korean wastewater treatment plants (Kim et al. 2020).

Ibidhi et al. (2021) estimated the country-specific national emission factor for CH_4_ emissions from enteric fermentation in dairy cattle in South Korea using the 2006 IPCC approach. They calculated the emission factor for different age groups of animals. With the developed emission factor for dairy cattle, it was determined that the South Korean dairy sector has the potential to reduce greenhouse gas emissions by approximately 97 × 10^3^ tons of CO_2_ equivalent per year, which corresponds to a reduction of 8% from the sector’s total emissions (Ibidhi et al. 2021).

Tongwane and Moeletsi (2021) conducted a study for 2019, which determined that South Africa produced 35.37 million tons of CO_2e_ emissions, including emissions from sources such as cattle, pasture, and grasslands. CH_4_ emissions from enteric fermentation accounted for 64.54% of the total emissions, followed by emissions from pasture, grasslands, and savannahs at 27.66%. Regarding emissions related to fertilizer management, 4.34% of the total emissions were attributed to nitrous oxide (N_2_O), and 3.45% were attributed to CH_4_ emissions (Tongwane and Moeletsi 2021).

Jeong et al. (2021) estimated the biogas production of a municipal wastewater treatment plant in South Korea with deep learning-based models. In the estimation results, the *R*^2^ value was obtained as 0.76 (Jeong et al. 2021).

Sun et al. (2021) analyzed that China reduced its total annual greenhouse gas by 2% due to biogas production from straw and its conversion (Sun et al. 2021).

Ocak and Acar (2021) evaluated the energy production potential of Turkey’s Marmara region and concluded that converting agricultural and animal wastes into biogas and then into electricity is economical (Ocak and Acar 2021).

Huo et al. (2021) estimated CO_2_ emissions from China’s agricultural biomass conversion based on life cycle assessment. They predicted the potential of agricultural biomass to replace fossil energy and reduce emissions under three scenarios, considering resource endowment and bioenergy potential of crop straw and livestock manure (Huo et al. 2021).

Ludlow et al. (2021) evaluated the potential of converting organic waste into energy using lower heating values in Chile and found that this corresponded to 3.3% of the annual energy demand (Ludlow et al. 2021).

Zubir et al. (2022) utilized livestock data from Malaysia from 2010 to 2019 to estimate greenhouse gas emissions from different animal species. In the livestock sector, poultry, pigs, non-dairy cattle, and goats were predominant. Non-dairy cattle were found to be the main contributor to CH_4_ emissions, accounting for 73.91% of enteric fermentation emissions. Regarding CH_4_ emissions from manure management, pigs accounted for 61.49%, while poultry accounted for 26.24%. Regarding direct N_2_O emissions from manure management, poultry contributed 63.25%, and non-dairy cattle accounted for 20.79%. Enteric fermentation was noted to have the largest share in total CO_2_ equivalent emissions, surpassing 50% (Zubir et al. 2022).

Fajobi et al. (2022) comprehensively examined the studies in the literature addressing the effects of different biomass sources used in biogas production on biogas yield with different techniques. It evaluated artificial intelligence’s applicability in modeling and optimizing the anaerobic digestion process for different parameters. They used the fuzzy logic-based ANFIS method to estimate biogas yield (Fajobi et al. 2022).

Hörtenhuber et al. (2022) examined Austria’s greenhouse gas emissions from livestock and the effects of livestock farming on climate for the years 1990 and 2019. It has been shown that CH_4_ reduction from livestock reduces total CO_2_ emissions by 16% (Hörtenhuber et al. 2022).

Senocak and Guner Goren (2023) made a 5-year prediction with the support vector machine algorithm for animal, agricultural, and municipal solid wastes, which are biomass resources in Denizli province of Turkey (Senocak and Guner Goren 2023).

Sharafi et al. (2023) measured the long-term energy efficiency of Iran’s significant crops between 1970 and 2019. Greenhouse gas emissions were modeled with machine learning algorithms using 17 agricultural products in five main categories as input parameters (Sharafi et al. 2023).

Zhang et al. (2023) estimated the number of biomass resources that can be used in energy in 2020 by using specific parameters and coefficients. They also evaluated the potential to reduce CO_2_ emissions using biomass energy depending on its life cycle (Zhang et al. 2023).

Liu et al. (2023) estimated the biogas potential of agricultural waste in Hubei Province in China and evaluated the environmental and economic impact of CO_2_ reduction (Liu et al. 2023).

Nehra and Jain (2023) examined the estimation of animal-based biomass potential and the reduction of greenhouse gas emissions in rural Haryana, India. They stated that biomass energy production could prevent emissions of approximately 1707 to 3583 million kg/year (Nehra and Jain 2023).

Ceylan et al. (2023) developed a hybrid optimization model for Manisa, Turkey, utilizing a neuro-regression approach to determine the optimal biogas power plant location (Ceylan et al. 2023).

Heydari et al. (2023) studied the optimal design of a renewable wind/solar/biomass hybrid system for grid-independent applications in Iran by comparing the performance of genetic algorithms and particle swarm optimization. Simulation results showed that the photovoltaic/biomass system is cost-effective, and particle swarm optimization yields better results (Heydari et al. 2023).

It is possible to predict environmental quantities using boosting algorithms, an ML algorithm. By combining many weak models, these algorithms produce a single robust model. In most cases, the real-time dataset is nonlinear. Consequently, if a model cannot accurately define the dataset values, it will become underfitted and biased. In this case, boosting algorithms are necessary to reduce bias.

In this study, Adaptive Boosting (AdaBoost), Gradient Boosting, and eXtreme Gradient Boosting (XGBoost), which are popular boosting algorithms in the literature, were used to estimate the amount of biogas and CH_4_ emissions from animal sources. Biogas and CH_4_ quantities were calculated for 81 provinces of Turkey based on cattle, small ruminants, and poultry numbers. To determine the theoretical biogas and CH_4_ quantities, general and specific information about cattle, small ruminants, and poultry was used, along with data about animal age, number, breed, weight, and waste quantity. A data set was created to carry out further analysis.

With Turkey taking part in the Paris Agreement, it has plans to increase renewable energy production and reduce greenhouse gas emissions with the preparations for the 2050 climate change strategy and 2030 action plan. It also aims to develop dynamics for using artificial intelligence methods in the country. In this sense, it is crucial to accurately estimate Turkey’s energy potential and emissions regarding animal husbandry-related research. Detailed examination and modeling of the livestock-based biogas potential in Turkey with boosting algorithms and energy and emission estimates based on this potential for the coming years can give researchers and policy planners ideas. In this study, biogas potential and CH_4_ emission values in cross-validation and time series format have been converted to log10 and predicted with boosting algorithms for regression analysis. Boosting algorithms were preferred because they reduce bias and make more successful predictions than classical ML algorithms.

The proposed study includes two different analyses:

- The first one includes the biogas potential and CH_4_ emission values of each province in Turkey for the years 2004–2019 and the forecasts of each of these values for the years 2020–2021 with the boosting algorithms,
- The second one uses the same values for 2004–2021 and makes predictions for 2022–2024.

The novelty and contribution are (i) animal-based biogas potential and CH_4_ emission were estimated by boosting algorithms using the unique identifier of the provinces and year information; (ii) the appropriate boosting model that can make predictions for all provinces of Turkey for the coming years has been created.

This study is presented in four sections and organized as follows: In this section, statistical information about the potential of renewable energy, animal husbandry, emissions, and biogas in Turkey is presented, and a literature review is included. In the “Materials and methods” section, the parameters used in theoretical biogas potential and CH_4_ emission calculations and the machine learning methods recommended for estimation are explained in detail. In the third section, the experimental results of machine learning algorithms are compared under two different scenarios, and future predictions are made. In the last section, conclusions and recommendations are made.

## Materials and methods

### Theoretical biogas potential calculation

**Fig. 1:**
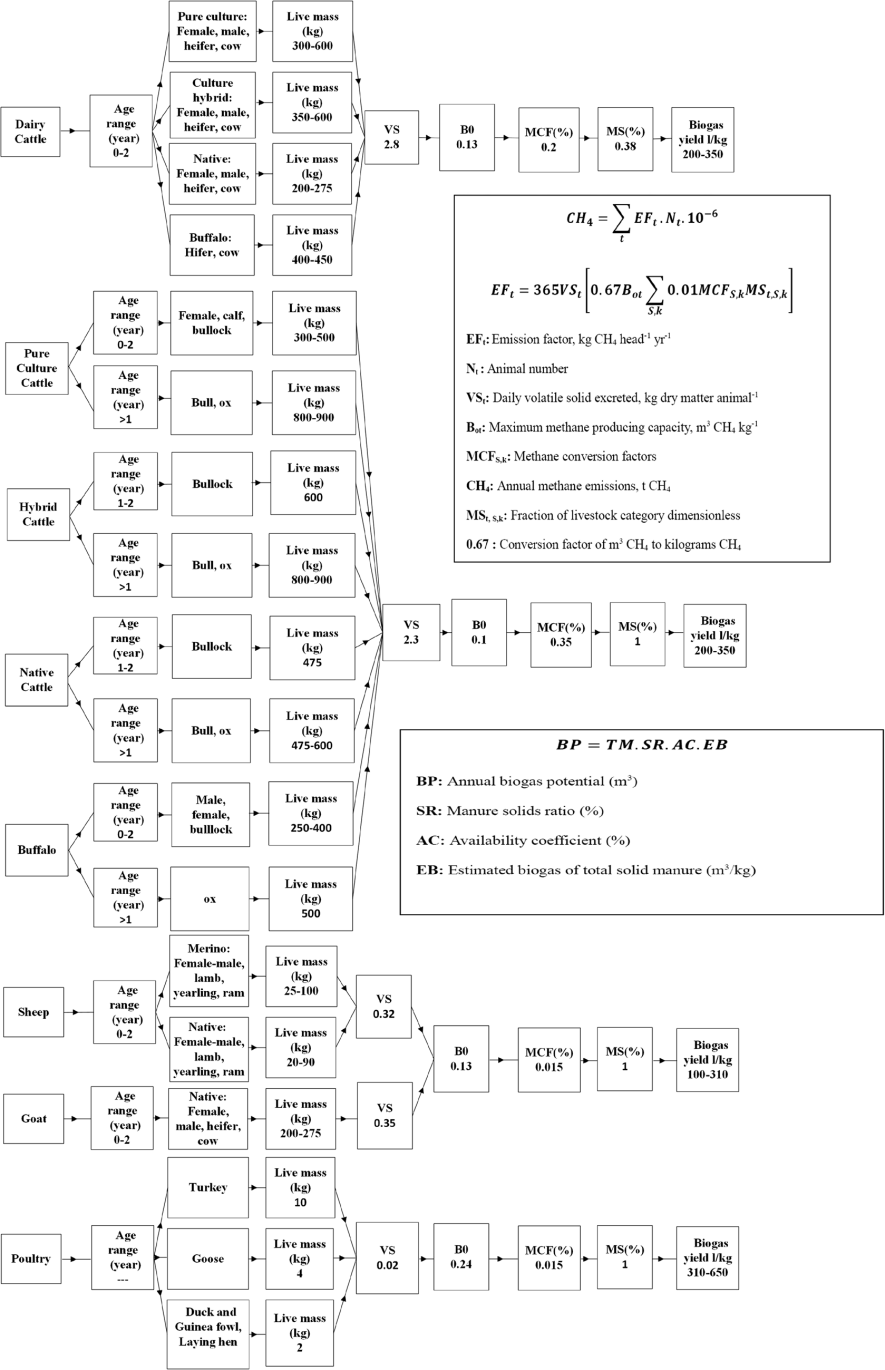
Formulas and parameters for biogas and CH_4_ emission (tier1, tier2 approaches)

Between 2004 and 2021, CH_4_ production through biogas was determined for each of the 81 provinces in Turkey using the animal population data of various animal species. Each animal species was categorized separately based on age, gender, and weight. Live weight values specific to animal species and breeds were obtained from farms within the provinces to determine the amount of manure. Since no representative value was available for manure production in Turkey, the percentages of live weight values obtained from the literature were used. The percentages used were 6% for cattle, 5% for small ruminants, and 4% for poultry. The daily fresh manure values were calculated separately for each province and district based on the age and species of cattle and small ruminants and separately for poultry using these percentages. The amount of animal waste varies depending on feeding practices, climate conditions, and reproductive types. The usability coefficients for each animal species were 50% for cattle, 13% for small ruminants, and 99% for poultry. Figure 1 provides the details of the animal species along with the parameters (VS, B_0_, MCF, MS) used in the calculation of CH_4_. These parameters were utilized in the tier2 approach for CH_4_ estimation (Avcioǧlu and Türker 2012; Dong et al. 2006).

If animal manure is not effectively managed and processed in a biogas production system, it can result in the uncontrolled release of CH_4_ gas into the atmosphere. The agricultural and livestock industries are significant contributors to greenhouse gas emissions, and the improper handling of animal waste exacerbates this issue. The release of CH_4_, a potent greenhouse gas, further contributes to global warming and climate change. Therefore, proper collection and treatment of animal manure within biogas systems are essential for mitigating greenhouse gas emissions and promoting sustainable agricultural practices (Riaño and García-González 2015).

CH_4_ emissions are calculated using different methods, with tier1 and tier2 being the most commonly used approaches. In tier1, a simple calculation is employed, multiplying the number of animals in each category by the emissions factor per animal. Tier2, on the other hand, is a more advanced method implemented in most developed countries. It involves considering various parameters specific to each animal species. The Intergovernmental Panel on Climate Change (IPCC) provides assumed emission factors for each livestock category, considering the average annual temperature. These emission factors reflect the range in manure volatile solids content and the application of manure management practices in different regions. They have been evaluated based on the annual temperature for each climatic region. By utilizing tier2 methodology and considering these emission factors, a more accurate estimation of CH_4_ emissions from livestock can be obtained. This approach allows for a more comprehensive assessment of the environmental impact of livestock farming and helps develop targeted strategies for reducing greenhouse gas emissions in the agricultural sector.

The CH_4_ emissions were calculated using different approaches. For the tier1 approach, the formula provided in Fig. 1 and the emission factors specific to each region, as listed in Table 10.11 of IPCC-2006 (Dong et al. 2006), were utilized. This method involves multiplying the emission factor by the number of animals in each category. On the other hand, the tier2 approach employed the formulas depicted in Fig. 1, along with the parameter values specified in Table 1. This more advanced method considers additional parameters and variables associated with each animal species. Tier1 and tier2 approaches allow for a comprehensive calculation of CH_4_ emissions. These approaches provide valuable insights into the environmental impact of livestock farming and assist in devising effective strategies for mitigating greenhouse gas emissions in the agricultural sector. The IPCC approach was used to calculate CH_4_ emissions from enteric and manure in the dairy cow system (Baek et al. 2014) and for values from beef cattle (Chen et al. 2020). In addition, an application was made for Korea in agricultural biomass calculation (Shin et al. 2016). While the greenhouse gas detection studies carried out using the tier1 approach include Ngwabie et al. (2018), the tier2 approach was used in the studies of Herrera et al. (2021) and Basak et al. (2022). While Khan et al. (2021) examined Pakistan’s biogas production potential from animal manure (Khan et al. 2021), Şenol et al. (2021) carried out studies on calculating Turkey’s biogas potential until 2030 (Şenol et al. 2021).

**Table 1:** The performance scores for modeling animal-based biogas potential for 2004–2021

Analysis	Algorithm	Train			Test/prediction		
		RMSE	MAE	*R*^2^	RMSE	MAE	*R*^2^
Cross-validation (2004–2019)	Gradient Boosting	0.259 ± 0.004	0.212 ± 0.003	0.8479 ± 0.0044	0.285 ± 0.016	0.232 ± 0.012	0.8134 ± 0.0239
	XGBR	0.036 ± 0.002	0.025 ± 0.001	0.9971 ± 0.0003	0.106 ± 0.013	0.071 ± 0.006	**0.9741 ± 0.0058**
	AdaBoost	0.500 ± 0.004	0.427 ± 0.004	0.4342 ± 0.0079	0.511 ± 0.015	0.434 ± 0.010	0.4040 ± 0.0447
Prediction (2020–2021)	Gradient Boosting	0.254	0.207	0.8543	0.247	0.201	0.8439
	XGBR	0.036	0.026	0.9970	0.067	0.054	**0.9883**
	AdaBoost	0.496	0.424	0.4432	0.500	0.422	0.3567

### Creation of the biogas and CH_4_ quantities data set

In this study, the biogas and CH_4_ (tier1 and tier2 approaches) quantities for each of the 81 provinces in Turkey for the years 2004–2021 were calculated based on the data obtained from the Turkish Statistical Institute (TUIK) regarding the number of cattle, small ruminants, and poultry (TUIK 2022). The calculation used general and specific information about cattle, small ruminants, and poultry and data on animal age, count, breed, weight, and waste quantity to determine the theoretical biogas and CH_4_ quantities. The biogas potential and CH_4_ emissions (tier1 and tier2 approach) of each animal category in all provinces of Turkey for 2004–2021 were theoretically calculated, and a data set was created. While the biogas potential and CH_4_ emissions are the target values, only the unique identifier of the provinces and year information is the input value. This way, training and prediction can be performed in a time series format. The dataset contains 1458 samples for 18 years for 81 provinces in Turkey. For scenario-1, 1296 data from 2004 to 2019 were used for training, and 162 data from 2020 to 2021 were used for testing. For scenario-2, all 1458 data from 2004 to 2021 were used for training, and the years 2022–2024 were predicted.

During regression analysis, log transformations are used to reduce the distance between data points and help develop a better model. Due to the extensive range of values and sometimes outliers included in the dataset, this may be the case. In log10 transformations, base 10 is used to determine the logarithm. In addition to being more readily interpreted or checked, common logarithms are more straightforward to comprehend (Zhang et al. 2022).

This study transformed biogas potential and CH_4_ emission values to log10 because of their wide range of values in training boosting algorithms.

### Ensemble learning for prediction

Ensemble learning in ML refers to an ensemble of base learners working together to make a more accurate final prediction. As a result of high variances and biases, a single weak learner might not perform well alone. However, by combining weak learners, a strong learner can be created. It is possible to improve model performance by combining weak learners in this manner. Consequently, a problem can be solved more effectively by combining several ML algorithms. Ensemble learning algorithms train multiple models on the data and then combine their predictions to produce the final result. In regression problems, this combination is done by taking the average of the predictions. There are various methods for model averaging, including one-step and iterative weighted parameter estimation. The objective is to increase the true conditional mean of the dependent variable provided by the predictors’ prediction accuracy. This means adjusting the model’s predictions under different conditions or values of the predictors to match the actual average values of the dependent variable more closely. The idea is to develop a model that can more accurately predict the conditional mean by capturing the relationships between the predictors and the dependent variable.

The total prediction error of an ML model is composed of bias and variance errors. Bias measures how far off the model’s predictions are from the actual values. If the bias is high, the model does not accurately capture the relationship between the input and output variables and may need to be more complex. Variance measures how much the model’s predictions change when trained on different subsets of the data. If the variance is high, the model is overfitting to the training data and may need to be simplified or trained on more data.

Ensemble learning methods such as bagging and boosting differ primarily in how they are trained. A bagging method involves training weak learners in parallel, whereas a boosting method involves training them sequentially.

The boosting technique is used in ensemble models to improve the generalization of a weak learning model, such as decision trees. Better prediction is obtained compared to the single weak learner using methods like majority voting in classification problems or a linear combination of weak learners in regression problems.

In boosting, multiple weak learners are combined to create a strong learner. A boosting algorithm differs from a bagging algorithm because it aims to reduce bias rather than variance. Boosting involves adjusting the next model’s weighting based on the previous model’s performance so that new subsets will contain elements that previous models had misclassified. The purpose of boosting is to improve the performance of a relatively simple classifier with a high bias rate. It is necessary to train each of the base classifiers sequentially. As a result of high bias, the model fails to capture the essential features of data because the assumptions it makes are too basic, and boosting algorithms are used to reduce high bias.

The algorithm for boosting is as follows:

1. Initialize the dataset and assign the weights of all data points to be equal.
2. Train a weak learner on the weighted data and compute the error between predicted and actual values.
3. Increase the weights of the data points with significant errors and decrease the weights with minor errors.
4. The algorithm passes the updated weights to the next learner.
5. Steps 2–4 should be repeated until the training error is less than a predetermined threshold or for a fixed number of iterations.
6. The results from each weak learner are combined.

Boosting is a widely used technique for solving classification and regression problems. The most popular boosting algorithms in the literature are Adaboost, Gradient Boosting, and XGBoost. In this study, boosting algorithms were customized for regression analysis to estimate biogas potential and CH_4_ emission values in cross-validation and time series format.

#### Adaptive boosting

AdaBoost is the first boosting algorithm introduced by Freund and Schapire (1997) and combines weak learners to create a strong learner. It is known as adaptive boosting because each instance receives an updated set of weights, with higher weights given to incorrectly classified instances. It combines weak classifiers iteratively trained on incorrectly classified samples from the previous iteration into a strong classifier (Ganaie et al. 2022). This algorithm can also be used for regression problems.

As a first step in the AdaBoost algorithm, ${\omega }^{\left(i\right)}$ which is the weight of each sample, is assigned an initial value of (1/*n*) equally. The first learner is then trained, and the weighted error rate is calculated. In Eq. (1), the weighted error rate of the *j*th learner is calculated.

1 $${r}_{j}=\frac{\sum_{\begin{array}{c}i=1\\ {\widehat{y}}_{j}^{\left(i\right)}\ne {y}^{\left(i\right)}\end{array}}^{m}{\omega }^{\left(i\right)}}{\sum_{i=1}^{m}{\omega }^{\left(i\right)}}$$

In Eq. (1), ${\widehat{y}}_{j}^{\left(i\right)}$ represents the *j*th learner prediction for *i*th sample. The weights of the learners are calculated according to Eq. (2).

2 $${\alpha }_{j}=\eta {\text{log}}\frac{1-{r}_{j}}{{r}_{j}}$$

In Eq. (2), $\eta$ is the learning rate. The AdaBoost algorithm then updates the weights of the incorrectly predicted examples to speed up the learning rate, as given in Eq. (3).

3 $${\omega }^{\left(i\right)}=\left\{\begin{array}{c}i=\mathrm{1,2},\dots ,m\\ {\omega }^{\left(i\right)}, {\widehat{y}}_{j}^{\left(i\right)}={y}^{\left(i\right)}\\ {\omega }^{\left(i\right)}{e}^{{\alpha }_{j}}, {\widehat{y}}_{j}^{\left(i\right)}\ne {y}^{\left(i\right)}\end{array}\right.$$

The weights of all samples are then normalized by dividing by $\sum_{i=1}^{m}{\omega }^{\left(i\right)}$. Finally, a new learner is trained with the updated weights, and the process continues until the termination criterion is reached (Géron 2019).

#### Gradient boosting

Gradient Boosting is a generic algorithm that sequentially assembles tree models. A generalization of the AdaBoost algorithm, gradient boosting allows any differentiable loss function. The difference between the predicted and actual values of the outcome variable is determined by fitting the tree to the loss function’s negative gradient. This allows it to optimize arbitrary differential loss functions (Friedman 2001). Gradient Boosting is an ensemble model that makes predictions by “boosting” the collection of subpar prediction models to create a more reliable model. The errors learned from previous base learners are the focus of this model’s training of the current base learner (Otchere et al. 2022).

#### Extreme gradient boosting

XGBoost approach is one of the most popular gradient-boosted decision tree implementations and can solve the sparse data problem. The training process is accelerated by hardware acceleration and parallel processing in XGBoost, an optimized Gradient Boosting implementation. Regularization, weighted quantile sketches, parallel learning blocks, cache awareness, and out-of-core computing capabilities are all provided by XGBoost. L1/L2 penalties are used for regularization to control overfitting. Utilizing the weighted quantile sketch algorithm, it can also handle sparse data sets. The main idea behind the XGBoost algorithm is to divide features and add trees to grow a tree continuously. The predicted value of the sample is calculated by adding up the scores corresponding to each tree if a prediction is generated for each sample after training. This score is determined using the characteristics of this sample, which correspond to a leaf node in each tree (Chen and Guestrin 2016; Khan et al. 2023). XGBoost Regressor (XGBR) is used for regression problems.

The objective function of the XGBR algorithm is given in Eq. (4), while the regularization term in this function is given in Eq. (5).

4 $$OBJ=\sum_{i=1}^{n}L\left({\widehat{y}}_{i},{y}_{i}\right)+\sum_{t=1}^{k}\Omega \left({f}_{t}\right)$$

5 $$\Omega \left(f\right)=\gamma T+\frac{1}{2}\lambda {\Vert \omega \Vert }^{2}$$

**Fig. 2:**
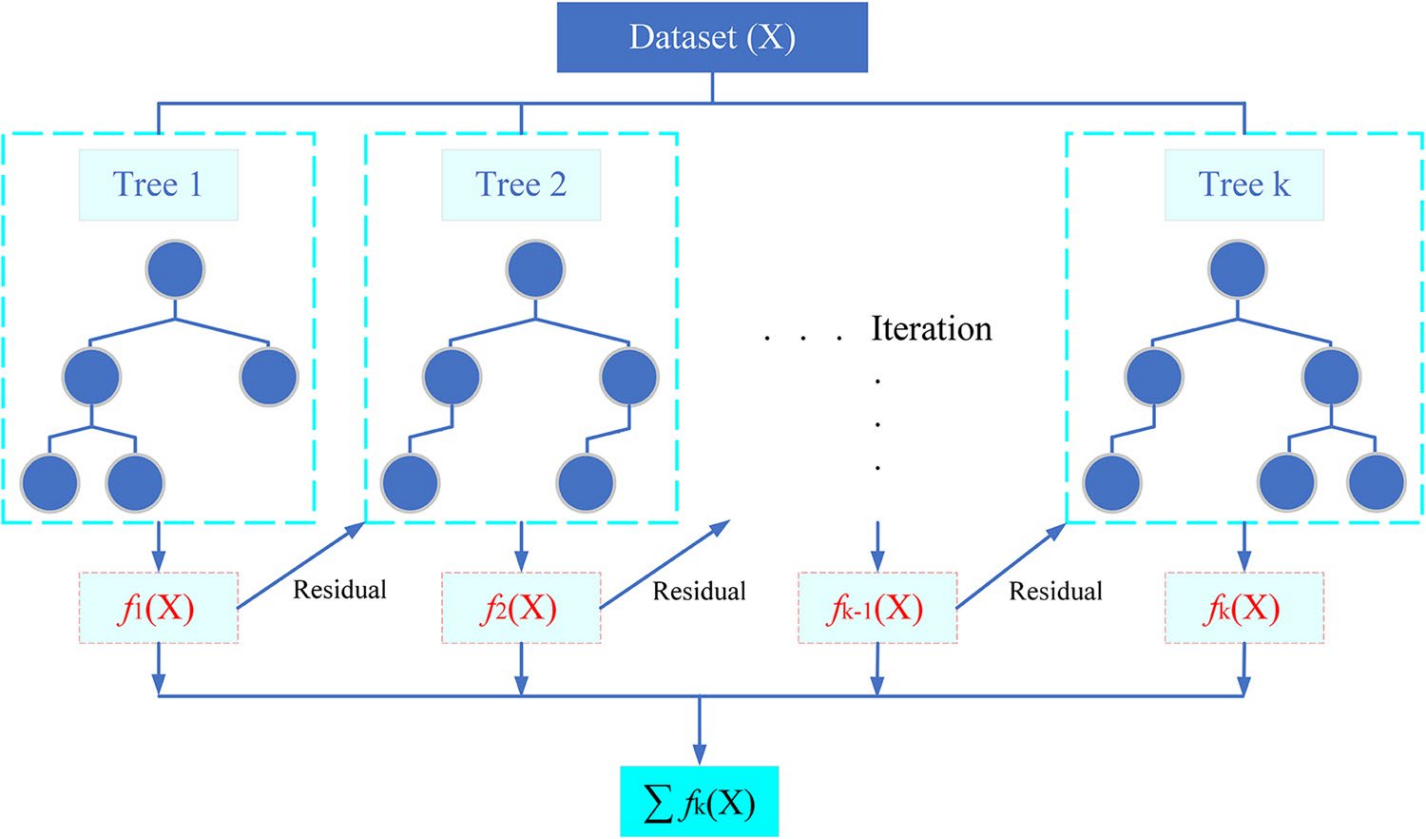
The structure of XGBR for biogas potential and CH_4_ emission prediction (${f}_{k}$: the predicted value of each tree)

In Eqs. (4) and (5), *L*(.) and *Ω*(.) refer to the loss function and the regularization term. The target value is *y*, and the predicted value is $\widehat{y}$, the number of samples is *n*, and the current sample is *i* in the loss function. *k* denotes the number of trees in the current model, *t* denotes the current tree, *T* denotes the total number of leaf nodes, and *ω* denotes the weight of each leaf. This term of regularization suppresses the complexity of the model that forms the objective function. Control parameters to prevent overfitting include *γ* and *λ*. The structure of XGBR is given in Fig. 2.

### Model evaluation

Testing the model on the test set after training should be conducted to determine its performance and generalizability. It is possible to use metrics for evaluating models in this context. An analysis of regression was conducted using the root mean square error (RMSE), mean absolute error (MAE), mean absolute percentage error (MAPE), and coefficient of determination (*R*^2^), which are commonly used metrics for regression analysis. Equation (6)–(9) provides the equations for these metrics (Hajabdollahi Ouderji et al. 2023).

6 $$RMSE=\sqrt{\frac{1}{n}\sum\nolimits_{i=1}^{n}{\left({y}_{i}-{\widehat{y}}_{i}\right)}^{2}}$$

7 $$MAE=\frac{1}{n}\sum\nolimits_{i=1}^{n}\left|{y}_{i}-{\widehat{y}}_{i}\right|$$

8 $$MAPE=\frac{1}{n}\sum\nolimits_{i=1}^{n}\left|\frac{{y}_{i}-{\widehat{y}}_{i}}{{y}_{i}}\right|$$

9 $${R}^{2}=1-\frac{\sum_{i=1}^{n}{\left({y}_{i}-{\widehat{y}}_{i}\right)}^{2}}{\sum_{i=1}^{n}{\left({y}_{i}-{\overline{y} }_{i}\right)}^{2}}$$

According to Eqs. (6)–(9), *y* represents the target value, $\widehat{y}$ represents the predicted value, $\overline{y }$ represents the mean of the target value, and *n* represents the sample size. MAPE is a relative measure based on percentage units instead of the variable’s units to compare prediction accuracy between time-series models.

Cross-validation in ML evaluates a model’s performance when it is applied to unseen data. Data is divided into multiple folds or subsets, and one fold serves as a validation set, while the other folds are used to train the model. The validation process is repeated several times, using different subsets of data each time. The model’s performance on unseen data is then estimated by averaging the results. In this study, tenfold cross-validation was also used on the training data in addition to future predictions.

The statistical significance of relationships between ML algorithms’ prediction values is assessed using statistical tests. The Wilcoxon rank-sum test confirms the inconsistencies between the model output and the actual value. If the *p* value of the model predictions is less than 0.05, the null hypothesis is rejected (Hayes et al. 2022).

### Boosting algorithm parameters

Default parameters in Sklearn and XGBoost libraries were used in all boosting algorithms. For Adaboost, the maximum number of predictors is 50, the learning rate is 1.0, and a linear loss function is used. For Gradient Boosting, the loss function is the square of the error, the learning rate is 0.1, the number of predictors is 100, the subsample rate is 1.0, the friedman_mse function is used to measure the quality of a split, min_samples_split is 2, min_samples_leaf is 1, and the maximum depth of individual regression predictors is 3. For XGBR, the booster is gbtree, which uses tree-based models, 0.3 step size reduction used in updating to avoid overfitting, gamma is 0, max_length is 6, min_child_weight is 1, 1.0 subsample ratio of training samples, sample_method is uniform, tree generation method is faster histogram optimized approximate greedy algorithm.

## Results and discussion

This study used Adaboost, Gradient Boosting, and XGBR, popular boosting algorithms in the literature, to estimate the amount of biogas and CH_4_ emissions from animal sources. The proposed study includes two different analyses:

- The first one includes the biogas potential and CH_4_ emission values of each province in Turkey for the years 2004–2019 and the forecasts of each of these values for the years 2020–2021 with the boosting algorithms (scenario-1),
- The second one uses the same values for 2004–2021 and makes predictions for 2022–2024 (scenario-2).

Python programming language (ver. 3.10) with Scikit-learn library with boosting algorithms and XGBoost library was used for the execution of the algorithms.

In scenario-1, data for the 2004–2019 period were analyzed with a tenfold cross-validation analysis. Then, 1296 data for these years were used for training, and 162 data for the period 2020–2021 were tried to be predicted. The performance scores of cross-validation and predictions for animal-based biogas potential are shown in Table 1.

Table 1 shows that the XGBR algorithm is more successful in training and test scores in cross-validation and 2020–2021 predictions.

**Fig. 3:**
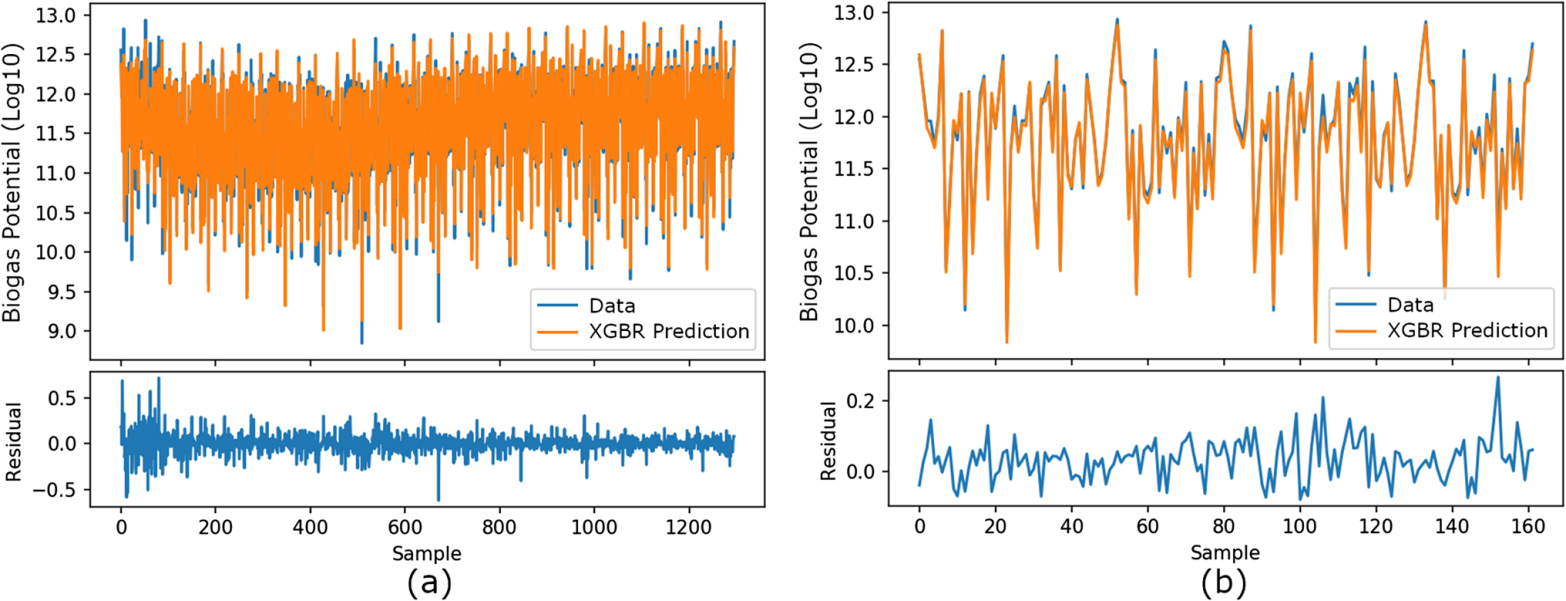
Comparison of XGBR’s predictions with the theoretical calculations of animal-based biogas potential: **a** tenfold cross-validation results and **b** prediction results for 2020–2021

In comparing XGBR’s predictions with the theoretical calculations of animal-based biogas potential, the tenfold cross-validation and prediction results for 2020–2021 are shown in Fig. 3a, b, respectively.

In Fig. 3a, the sample represents the record of each province for each year between 2004 and 2019, while in Fig. 3b, it represents each year between 2020 and 2021. In Fig. 3a, b, the value represents log10 of the animal-based biogas potential of the relevant record. In Fig. 3, the theoretical calculation values of 1296 samples of biogas potential between 2004 and 2019 vary between 8.848 and 12.934. Residual refers to the difference between theoretical calculation and predicted values in statistical analysis. In the residual graphs given in Fig. 3, the values in 2004 fluctuate between − 0.5 and 0.5, while the values for other years are monotonous. The residual graph of the 2020–2021 prediction shows that the values are monotonic and closer to 0.

**Fig. 4:**
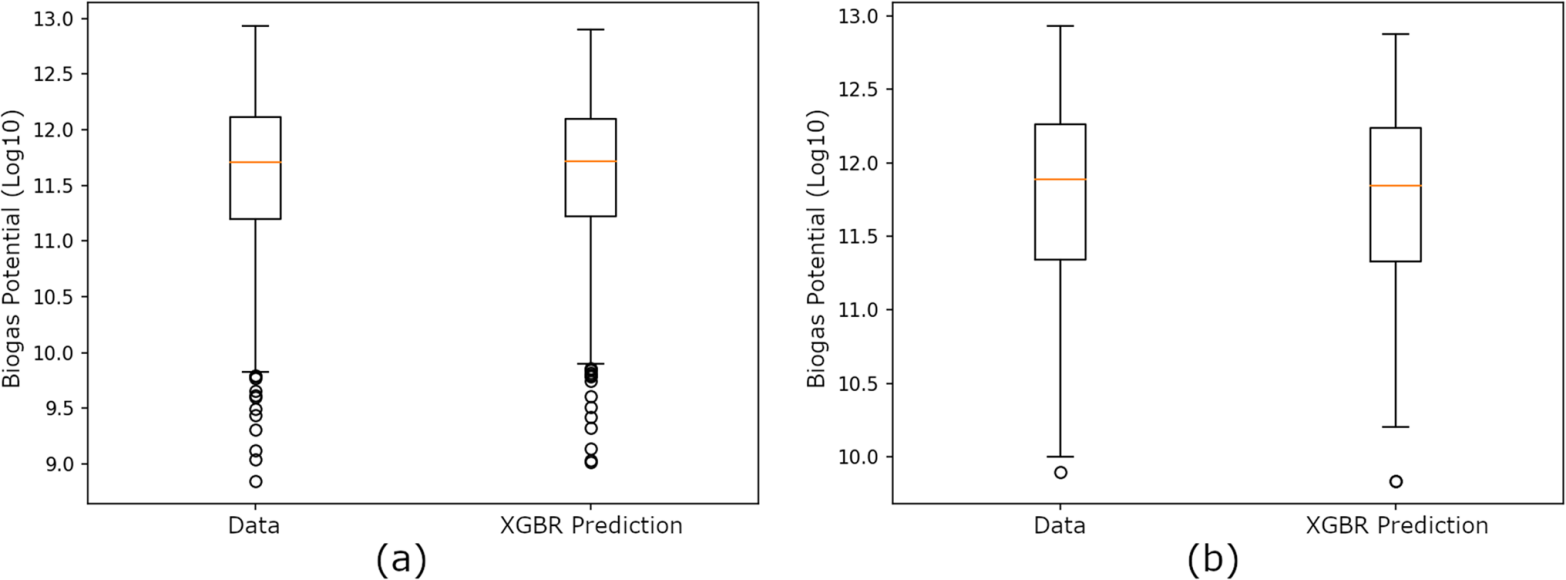
Box plots of XGBR’s predictions with the theoretical calculations of animal-based biogas potential: **a** tenfold cross-validation results and **b** prediction results for 2020–2021

As seen in Fig. 3, the XGBR predictions are consistent with the original curve, and the results can also be seen as a box plot in Fig. 4.

According to Fig. 4, the XGBR predictions (first quartile 11.20, median 11.71, and third quartile 12.05 for Fig. 4a, while first quartile 11.32, median 11.88, and third quartile 12.25 for Fig. 4b) overlap with the theoretical calculation values.

The performance scores of cross-validation and predictions for animal-based CH_4_ emissions are shown in Table 2.

**Table 2:** The performance scores for modeling animal-based CH_4_ emissions for 2004–2021

Approach	Analysis	Algorithm	Train			Test/prediction		
			RMSE	MAE	*R*^2^	RMSE	MAE	*R*^2^
Tier1	Cross-validation (2004–2019)	Gradient Boosting	0.213 ± 0.003	0.151 ± 0.002	0.9898 ± 0.0003	0.238 ± 0.029	0.165 ± 0.017	0.9855 ± 0.0057
		XGBR	0.042 ± 0.003	0.022 ± 0.001	0.9996 ± 0.0000	0.186 ± 0.056	0.080 ± 0.017	**0.9914 ± 0.0043**
		AdaBoost	0.321 ± 0.002	0.254 ± 0.002	0.9768 ± 0.0009	0.329 ± 0.025	0.259 ± 0.020	0.9716 ± 0.0113
	Prediction (2020–2021)	Gradient Boosting	0.214	0.151	0.9897	0.209	0.153	0.6552
		XGBR	0.047	0.024	0.9995	0.046	0.034	**0.9835**
		AdaBoost	0.324	0.254	0.9764	0.333	0.254	0.1220
Tier2	Cross-validation (2004–2019)	Gradient Boosting	0.200 ± 0.004	0.159 ± 0.003	0.7872 ± 0.0068	0.217 ± 0.020	0.173 ± 0.011	0.7441 ± 0.0280
		XGBR	0.028 ± 0.002	0.018 ± 0.001	0.9959 ± 0.0004	0.119 ± 0.036	0.062 ± 0.007	**0.9191 ± 0.0437**
		AdaBoost	0.350 ± 0.006	0.289 ± 0.005	0.3530 ± 0.0190	0.354 ± 0.019	0.289 ± 0.019	0.3196 ± 0.0749
	Prediction (2020–2021)	Gradient Boosting	0.205	0.162	0.7766	0.211	0.170	0.7647
		XGBR	0.030	0.020	0.9953	0.066	0.055	**0.9773**
		AdaBoost	0.347	0.288	0.3612	0.365	0.297	0.2955

Table 2 shows that the XGBR algorithm gives the best results with *R*^2^ values of 0.9914 and 0.9191 in the cross-validation analysis for CH_4_ emissions with tier1 and tier2 approaches, respectively. On the other hand, in terms of 2020–2021 predictions, it is seen that the XGBR algorithm gives the best results with *R*^2^ values of 0.9835 and 0.9773 in predicting CH_4_ emissions with tier1 and tier2 approaches, respectively.

**Fig. 5:**
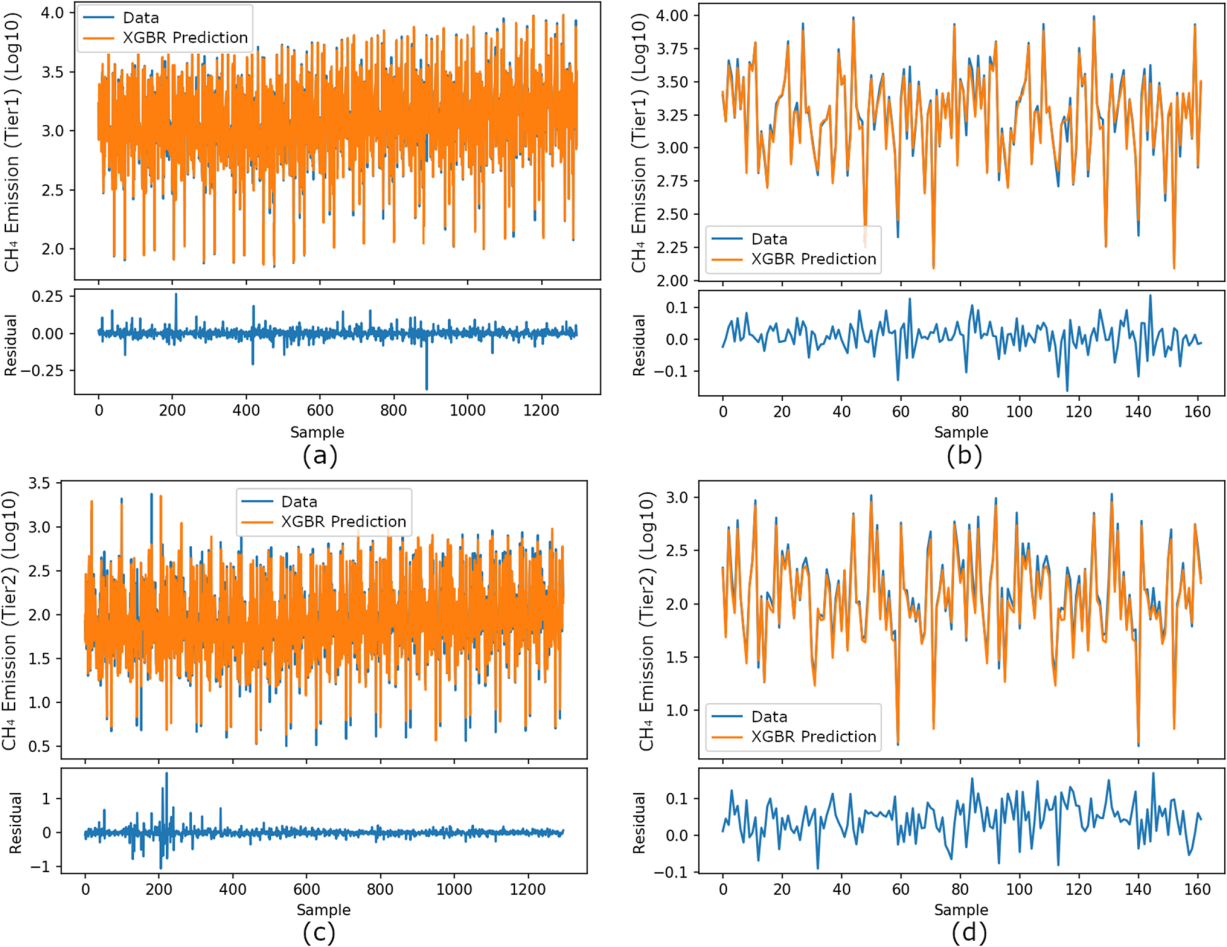
Comparison of XGBR’s predictions with the theoretical calculations of animal-based CH_4_ emissions: **a** tenfold cross-validation (tier1 approach), **b** prediction results for 2020–2021 (tier1 approach), **c** tenfold cross-validation (tier2 approach), and **d** prediction results for 2020–2021 (tier2 approach)

In comparing XGBR’s predictions with the theoretical calculations of animal-based CH_4_ emissions, the tenfold cross-validation and prediction results for 2020–2021 are shown in Fig. 5a–c and Fig. 5b–d, respectively.

In Fig. 5a–c, the sample represents the record of each province for each year between 2004 and 2019, while in Fig. 5b-–d, it represents each year between 2020 and 2021. In Fig. 5, the value represents log10 of the animal-based CH_4_ emissions of the relevant record.

In Fig. 5a, the theoretical calculation values of CH_4_ emissions (tier1 approach) for 16 years vary between 1.847 and 3.981. In the residual graphs given in Fig. 5a, b, the values for 2004–2019 fluctuate between − 0.25 and 0.25, while the 2020–2021 prediction values vary between − 0.1 and 0.1. In Fig. 5c, the theoretical calculation values of CH_4_ emissions (tier2 approach) vary between 0.502 and 3.380. In the residual graphs given in Fig. 5c, d, the values for 2006 fluctuate between − 1 and 1, while the values for other years monotonously approach zero. In the residual graph of the 2020–2021 prediction, the values vary between − 0.1 and 0.1.

**Fig. 6:**
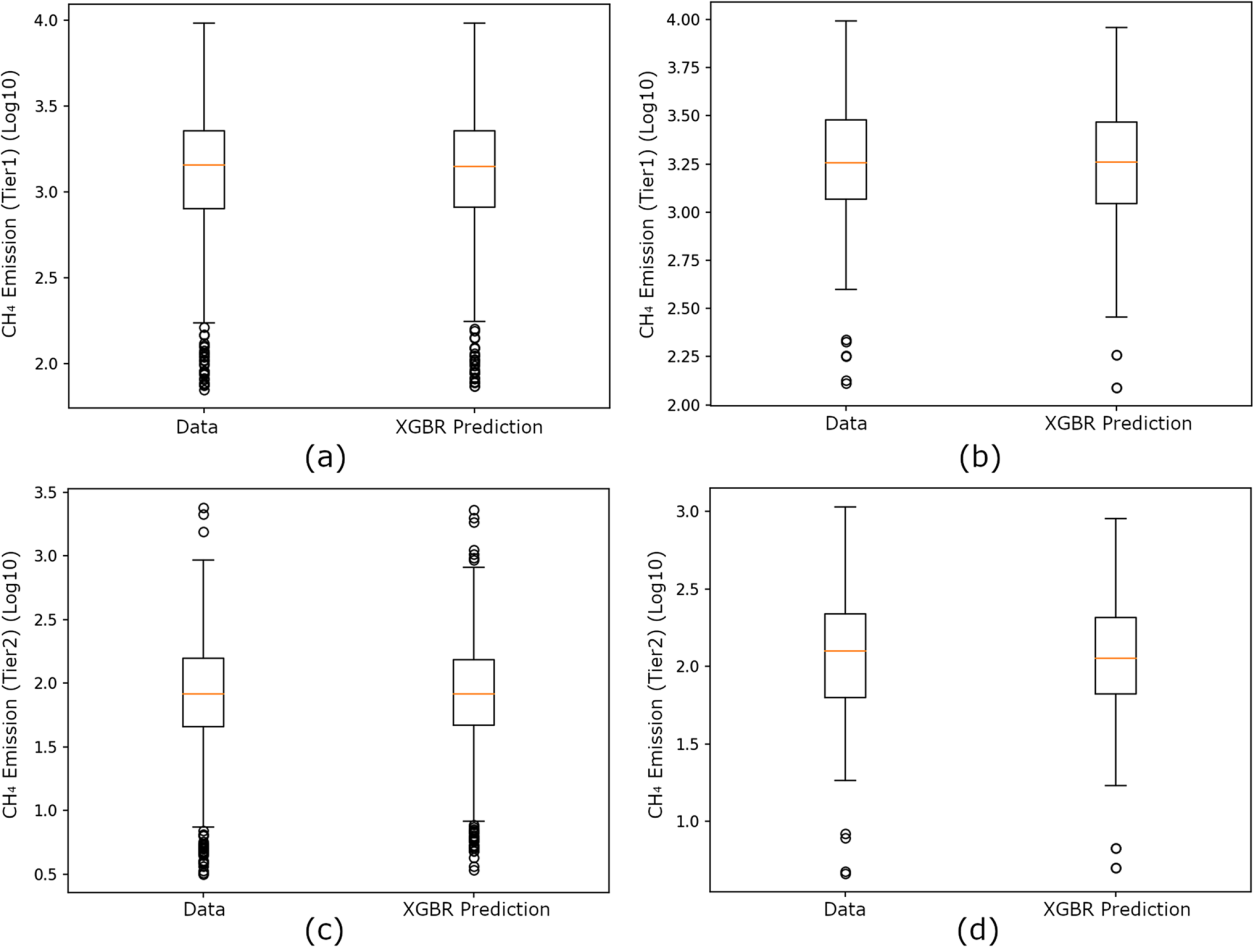
Box plots of XGBR’s predictions with the theoretical calculations of animal-based CH_4_ emissions: **a** tenfold cross-validation (tier1 approach), **b** prediction results for 2020–2021 (tier1 approach), **c** tenfold cross-validation (tier2 approach), and **d** prediction results for 2020–2021 (tier2 approach)

As seen in Fig. 5, the XGBR predictions are consistent with the original curve, and the results can also be seen as a box plot in Fig. 6.

In Fig. 6a, b, the XGBR predictions for CH_4_ emissions by tier1 approach (first quartile 2.88, median 3.15, and third quartile 3.31 for Fig. 6a, while first quartile 3.05, median 3.26 and third quartile 3.47 for Fig. 6b) overlap with the theoretical calculation values. According to Fig. 6c, d, the XGBR predictions for CH_4_ emissions by tier2 approach (first quartile 1.63, median 1.92, and third quartile 2.12 for Fig. 6c, while first quartile 1.80, median 2.10 and third quartile 2.33 for Fig. 6d) overlap with the theoretical calculation values.

Examining the error values as a percentage of the obtained models’ results can enable a more comfortable evaluation of the success of the models. Therefore, the comparison of the predictions and theoretical values of the boosting algorithms for animal-based biogas potential and CH_4_ emissions in terms of MAPE score are shown in Table 3.

**Table 3:** The MAPE scores for modeling animal-based biogas potential and CH_4_ emissions for 2004–2021

Category	Analysis	Algorithm	MAPE (%)	
			Train	Test/prediction
Biogas potential	Cross-validation (2004–2019)	Gradient Boosting	1.84 ± 0.03	2.02 ± 0.12
		XGBR	0.22 ± 0.01	**0.63 ± 0.05**
		AdaBoost	3.70 ± 0.04	3.77 ± 0.11
	Prediction (2020–2021)	Gradient Boosting	1.80	1.69
		XGBR	0.23	**0.46**
		AdaBoost	3.68	3.55
CH_4_ emissions (tier1)	Cross-validation (2004–2019)	Gradient Boosting	4.37 ± 0.05	4.73 ± 0.48
		XGBR	0.57 ± 0.03	**1.83 ± 0.24**
		AdaBoost	7.75 ± 0.06	7.88 ± 0.84
	Prediction (2020–2021)	Gradient Boosting	4.37	4.87
		XGBR	0.59	**1.07**
		AdaBoost	7.72	8.00
CH_4_ emissions (tier2)	Cross-validation (2004–2019)	Gradient Boosting	9.12 ± 0.25	9.91 ± 1.11
		XGBR	1.02 ± 0.05	**3.52 ± 0.48**
		AdaBoost	16.71 ± 0.54	16.50 ± 1.78
	Prediction (2020–2021)	Gradient Boosting	9.30	8.51
		XGBR	1.08	**2.78**
		AdaBoost	16.56	14.48

As seen in Table 3, the error rates of XGBR predictions for biogas potential were relatively low, approximately 0.63% and 0.46% for cross-validation and future prediction, respectively. For CH_4_ emission values, XGBR predictions are approximately 1.83% and 1.07% in tier1, while in tier2, they are approximately 3.52% and 2.78% for cross-validation and future prediction, respectively. Low percentage error values show that the XGBR algorithm is successful in modeling.

Table 4 contains statistical comparisons of the XGBR algorithm predictions of animal-derived biogas potential and CH_4_ emissions with theoretical values.

**Table 4:** Statistical comparisons of the XGBR algorithm predictions of biogas potential and CH_4_ emissions with theoretical values

Category	Algorithm	The Wilcoxon rank-sum test			
		Cross-validation analysis		2020–2021 prediction	
		*h*	*p*	*h*	*p*
Biogas potential	Gradient Boosting	-	0.032	+	0.117
	XGBR	** + **	**0.894**	** + **	**0.521**
	AdaBoost	-	0.000	-	0.000
CH_4_ emissions (tier1)	Gradient Boosting	+	0.700	+	0.165
	XGBR	** + **	**0.988**	** + **	**0.784**
	AdaBoost	-	0.023	-	0.000
CH_4_ emissions (tier2)	Gradient Boosting	+	0.344	+	0.120
	XGBR	** + **	**0.937**	** + **	**0.304**
	AdaBoost	-	0.000	-	0.000

The statistical test result is shown in Table 4 as *h* (“ + ,” accept; “ − ,” reject), and the test’s *p*-value is the probability that the null hypothesis is true. When the Wilcoxon rank-sum test results in Table 4 are examined, it is seen that there is no significant difference between the results of this model and the theoretical values at the significance level of *p* = 0.05.

Table 5 shows the theoretical values of biogas potential and CH_4_ emissions of five major provinces in Turkey for the year 2021 and the predictions of the XGBR algorithm.

**Table 5:** Theoretical values of biogas potential and CH_4_ emissions of 5 major provinces in Turkey for the year 2021 and the predictions of the XGBR algorithm

Province	Theoretical values (Log10)			XGBR predictions (Log10)		
	Biogas potential	CH_4_ emissions (tier1)	CH_4_ emissions (tier2)	Biogas potential	CH_4_ emissions (tier1)	CH_4_ emissions (tier2)
İstanbul	11.278	3.069	1.787	11.209	3.078	1.824
Ankara	12.084	3.696	2.818	11.960	3.605	2.705
İzmir	12.300	3.933	2.747	12.323	3.919	2.742
Bursa	11.850	3.445	2.567	11.894	3.404	2.450
Antalya	12.869	3.298	2.125	12.825	3.296	2.057

As seen in Table 5, the theoretical values of biogas potential and CH_4_ emissions for 2021 for five major provinces in Turkey and the predictions of the XGBR algorithm are close to each other.

In scenario-2, the values for 2004–2021 are used for training, and predictions are made for 2022–2024.

Table 6 shows the XGBR predictions for animal-based biogas potential and CH_4_ emissions of five significant provinces in Turkey for 2024.

**Table 6:** XGBR predictions for animal-based biogas potential and CH_4_ emissions for 2024

Year	Province	XGBR predictions (Log10)		
		Biogas potential	CH_4_ emissions (tier1)	CH_4_ emissions (tier2)
2024	İstanbul	11.279	3.080	1.810
	Ankara	12.055	3.652	2.806
	İzmir	12.309	3.929	2.757
	Bursa	11.869	3.411	2.552
	Antalya	12.866	3.321	2.122

According to the results in Table 6, the highest biogas potential among the five major provinces in Turkey in 2024 belongs to Antalya, while the highest CH_4_ emissions are estimated to be İzmir and Ankara for tier1 and tier2, respectively.

**Fig. 7:**
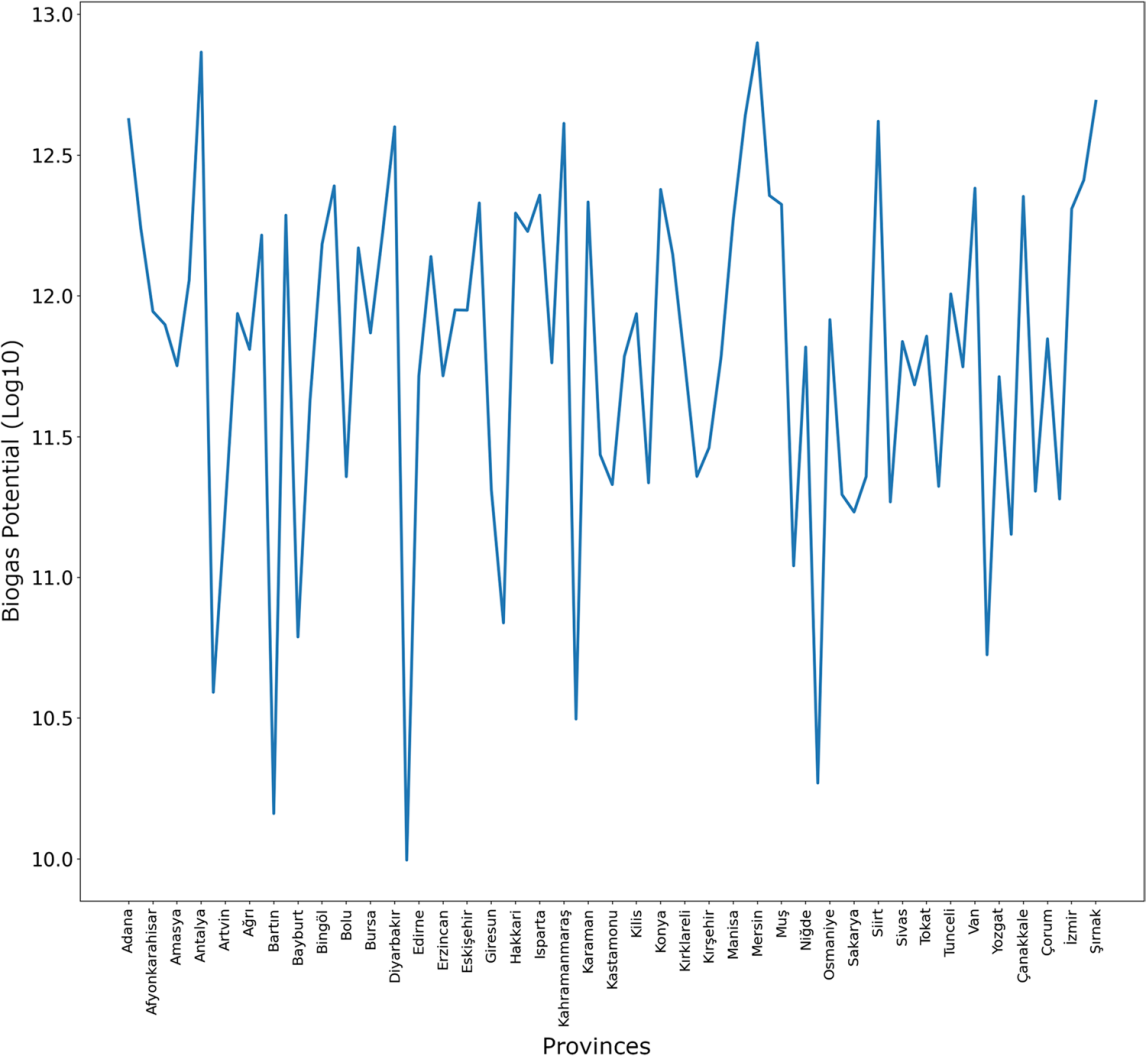
XGBR predictions for animal-based biogas potential for 2024

XGBR predictions for animal-based biogas potential for all provinces in Turkey in 2024 are shown in Fig. 7.

As seen in Fig. 7, the highest value in the 2024 animal-based biogas potential prediction for all provinces of Turkey belongs to Mersin province, while the lowest value belongs to Düzce province.

**Fig. 8:**
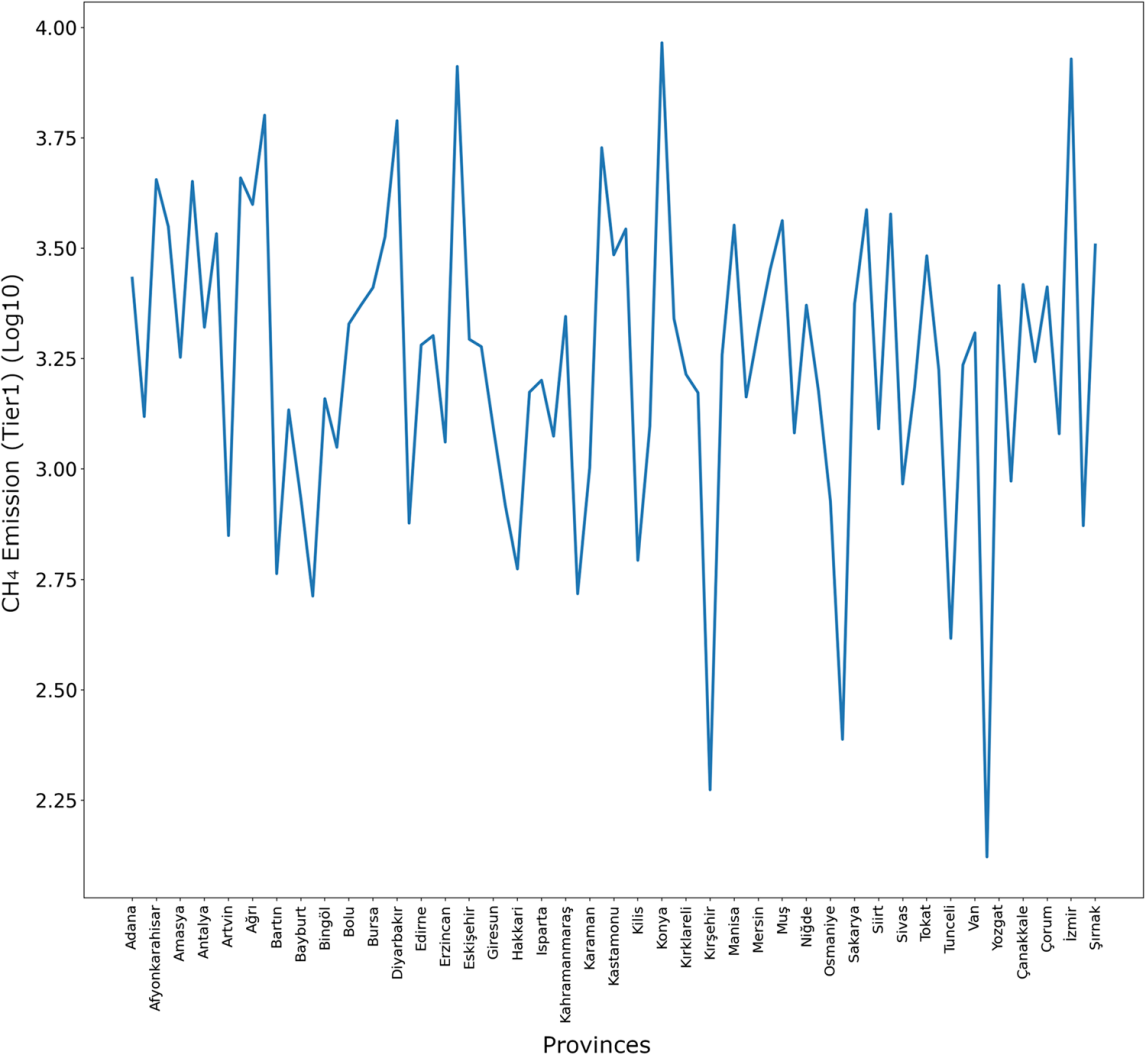
XGBR predictions for animal-based CH_4_ emissions (tier1 approach) for 2024

XGBR predictions for animal-based CH_4_ emissions (tier1 approach) for all provinces in Turkey in 2024 are shown in Fig. 8.

As seen in Fig. 8, the highest value in the animal-based CH_4_ emission (tier1 approach) prediction for 2024 in all provinces of Turkey belongs to Konya province, while the lowest value belongs to Yalova province.

**Fig. 9:**
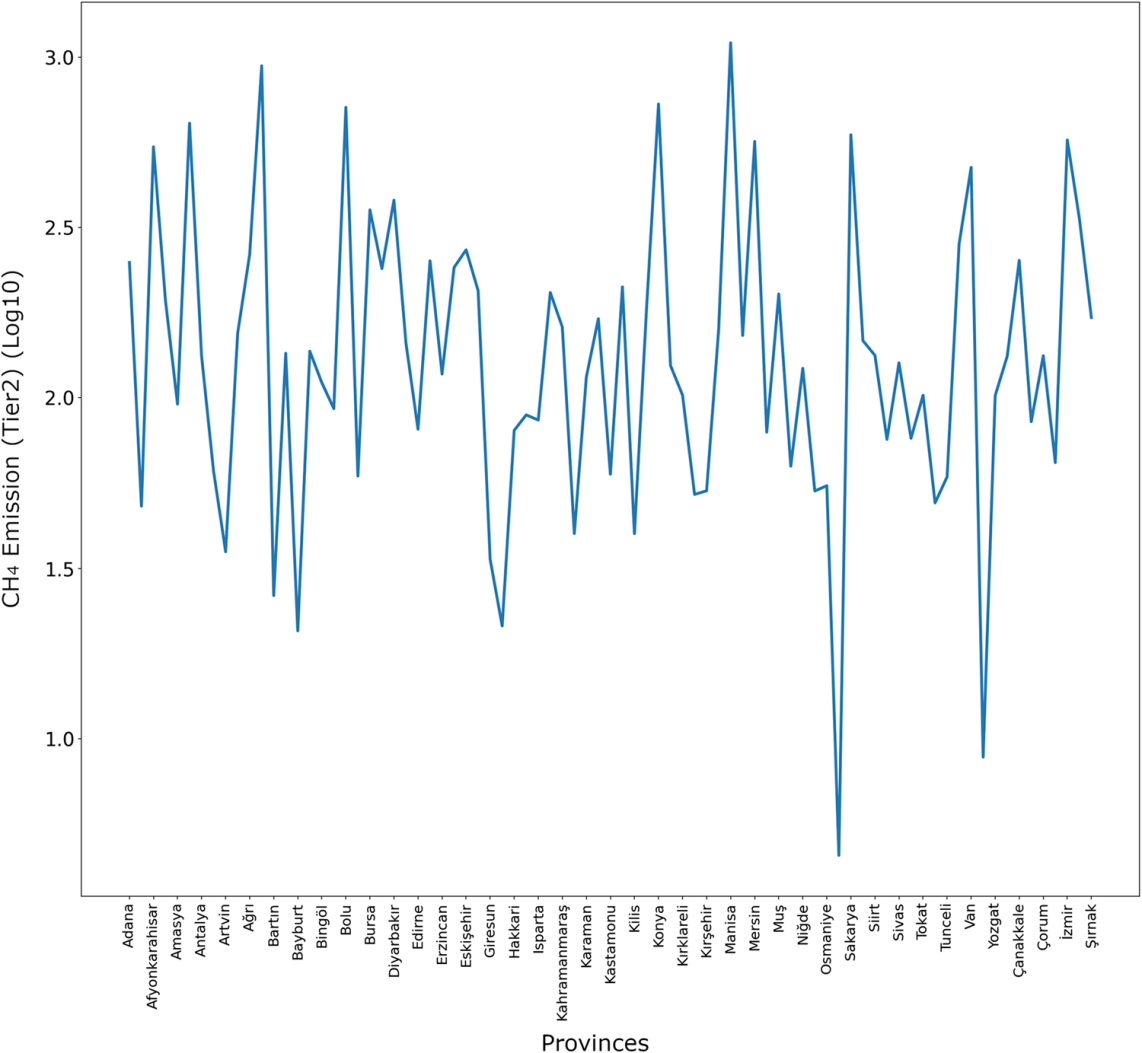
XGBR predictions for animal-based CH_4_ emissions (tier2 approach) for 2024

XGBR predictions for animal-based CH_4_ emissions (tier2 approach) for all provinces in Turkey in 2024 are shown in Fig. 9.

As seen in Fig. 9, the highest value in animal-based CH_4_ emission (tier2 approach) estimation for 2024 in all provinces of Turkey belongs to Manisa, while the lowest value belongs to Rize.

The greenhouse gas inventory for Turkey is calculated as 564.4 Mt CO_2e_ in 2021, increasing by 7.7% compared to 2020. Total greenhouse gas emissions per person increased by 0.4 tons compared to the previous year, reaching 6.7 tons of CO_2_ in 2021 (TUIK 2022). In this regard, it is thought that studies such as the current study will support Turkey’s 2050 climate change strategy and 2030 action plan preparations and the national contribution declaration, the Climate Change Directorate of the Ministry of Environment, Urbanization and Climate Change, and the relevant institutions within the scope of the United Nations Development Programme.

Although the study contains detailed information and analysis regarding animal husbandry in Turkey, it can only be considered limited to this area. However, it can also be improved in this context using different algorithms, parameters, etc. (e.g., agricultural wastes, geographical location, climatic conditions). The data in the study was obtained from the country’s statistical institution. These data-induced errors can also affect the applicability of the model. The information obtained as a result of the study will be statistically valuable.

## Conclusion

Emissions from the livestock sector have an essential place in climate change. Sustainable manure management and biogas production are crucial for countries to solve this problem. This study used boosting algorithms to investigate the animal-based biogas potential and CH_4_ emissions using tier1 and tier2 approaches in all Turkey provinces from 2004 to 2021. The XGBR algorithm was the most successful in predicting animal-based biogas potential and CH_4_ emissions, with MAPE ranging from 0.46 to 2.78%. The study also predicted the biogas potential of five major cities in Turkey for 2022–2024. The European Union aims to be a global role model in combating the climate crisis and achieving sustainable development goals. In this regard, countries want to reach climate neutral by 2050 with the Green Deal agreement, and it is thought that the prediction model proposed in this study can guide researchers for the coming years.

## Data Availability

All data generated or analyzed during this study are included in this published article.
